# Field-testing of primary health-care indicators, India

**DOI:** 10.2471/BLT.19.249565

**Published:** 2020-08-27

**Authors:** Devaki Nambiar, Hari Sankar, Jyotsna Negi, Arun Nair, Rajeev Sadanandan

**Affiliations:** a The George Institute for Global Health, 311–312, Third Floor, Elegance Tower, Plot No. 8, Jasola District Centre, New Delhi 110025, India.; b Baltimore, United States of America.; c ACCESS Health International, New Delhi, India.; d Health Systems Transformation Platform, New Delhi, India.

## Abstract

**Objective:**

To develop a primary health-care monitoring framework and health outcome indicator list, and field-test and triangulate indicators designed to assess health reforms in Kerala, India, 2018–2019.

**Methods:**

We used a modified Delphi technique to develop a 23-item indicator list to monitor primary health care. We used a multistage cluster random sampling technique to select one district from each of four district clusters, and then select both a family and a primary health centre from each of the four districts. We field-tested and triangulated the indicators using facility data and a population-based household survey.

**Findings:**

Our data revealed similarities between facility and survey data for some indicators (e.g. low birth weight and pre-check services), but differences for others (e.g. acute diarrhoeal diseases in children younger than 5 years and blood pressure screening). We made four critical observations: (i) data are available at the facility level but in varying formats; (ii) established global indicators may not always be useful in local monitoring; (iii) operational definitions must be refined; and (iv) triangulation and feedback from the field is vital.

**Conclusion:**

We observe that, while data can be used to develop indices of progress, interpretation of these indicators requires great care. In the attainment of universal health coverage, we consider that our observations of the utility of certain health indicators will provide valuable insights for practitioners and supervisors in the development of a primary health-care monitoring mechanism.

## Résumé

**Objectif:**

Mettre au point un cadre de suivi des soins de santé primaires ainsi qu'une liste d'indicateurs de résultats en santé. Tester sur le terrain et recouper les indicateurs conçus pour évaluer les réformes sanitaires dans le Kerala, en Inde, en 2018 et 2019.

**Méthodes:**

Nous avons utilisé une méthode de Delphes modifiée pour élaborer une liste de 23 indicateurs destinés à surveiller les soins de santé primaires. Nous avons eu recours à une technique d'échantillonnage aléatoire en grappes à plusieurs degrés pour sélectionner un district dans chacun des quatre groupes de districts, puis pour choisir à la fois un centre de médecine familiale et un centre de soins de santé primaires dans chacun des quatre districts. Nous avons testé les indicateurs sur le terrain et les avons recoupés à l'aide des données des établissements, ainsi que d'une enquête de population réalisée auprès des ménages.

**Résultats:**

Nos données ont montré des similarités entre les données des établissements et celles de l'enquête dans le cas de certains indicateurs (tels que l'insuffisance pondérale à la naissance et les services de vérification des antécédents). Néanmoins, d'autres indicateurs ont affiché des divergences (notamment au niveau des maladies diarrhéiques aiguës chez les enfants de moins de 5 ans et du dépistage de l'hypertension artérielle). Nous avons formulé quatre observations critiques: (i) les données sont disponibles au sein des établissements, mais sous différents formats; (ii) les indicateurs mondiaux prédéterminés se révèlent parfois inutiles dans le cadre d'un suivi à l'échelle locale; (iii) les définitions opérationnelles doivent être affinées; et enfin, (iv) il est indispensable de procéder à un recoupement des informations et de récolter les avis sur le terrain.

**Conclusion:**

Nous avons constaté que, même si les données peuvent être exploitées pour mettre au point des indices de progrès, interpréter ces indicateurs requiert beaucoup d'attention. En vue d'instaurer une couverture maladie universelle, nous estimons que nos observations relatives à l'utilité de certains indicateurs de santé fourniront des renseignements précieux aux praticiens et aux superviseurs dans le développement d'un mécanisme de suivi des soins de santé primaires.

## Resumen

**Objetivo:**

Elaborar un marco de supervisión de la atención primaria de salud y una lista de indicadores sobre los resultados en la salud, así como realizar ensayos de campo y triangular los indicadores previstos para evaluar las reformas sanitarias en Kerala, India, 2018-2019.

**Métodos:**

Se aplicó un método Delphi modificado para elaborar una lista de indicadores que incluye 23 elementos para supervisar la atención primaria de salud. Además, se empleó una técnica de muestreo aleatorio por conglomerados de etapas múltiples para seleccionar un distrito de cada uno de los cuatro conglomerados de distritos y, a continuación, se seleccionó una familia y un centro de atención primaria de cada uno de los cuatro distritos. Se realizaron ensayos de campo y se triangularon los indicadores mediante el uso de datos de los centros y una encuesta domiciliaria basada en la población.

**Resultados:**

Los datos obtenidos revelaron similitudes entre los datos de los centros y los de las encuestas para algunos indicadores (por ejemplo, el peso bajo al nacer y los servicios de control previo), así como diferencias para otros (por ejemplo, las enfermedades diarreicas agudas en niños menores de 5 años y la evaluación de la presión arterial). Se formularon cuatro observaciones críticas: i) los datos están disponibles a nivel de los establecimientos, pero en distintos formatos; ii) los indicadores globales establecidos no siempre son útiles para realizar una vigilancia local; iii) las definiciones operativas se deben perfeccionar; y iv) la triangulación y las observaciones en el terreno son vitales.

**Conclusión:**

Se observa que, si bien los datos se pueden usar para elaborar índices de progreso, la interpretación de estos indicadores requiere gran atención. Se cree que las observaciones obtenidas sobre la utilidad de ciertos indicadores de salud permitirán a los profesionales y a los supervisores comprender mejor el desarrollo de un mecanismo de vigilancia de la atención primaria de salud para lograr la cobertura sanitaria universal.

## ملخص

الغرض وضع إطار لرصد الرعاية الصحية الأولية، وقائمة مؤشرات النتائج الصحية، واختبار ميداني، ومؤشرات ثلاثية مصممة لتقييم الإصلاحات الصحية في ولاية كيرالا، في الهند الفترة 2018 و2019.

الطريقة استخدمنا أسلوب Delphi معدل لتطوير قائمة مؤشرات مكونة من 23 عنصرًا لرصد الرعاية الصحية الأولية. استخدمنا أسلوبًا عنقوديًا عشوائيًا متعددة المراحل لاختيار منطقة واحدة من كل أربع مجموعات للمناطق، ثم اخترنا أسرة ومركزًا صحيًا أوليًا من كل منطقة من المناطق الأربع. قمنا باختبار المؤشرات ميدانيًا وتقسيمها بشكل ثلاثي، باستخدام بيانات المرفق، ومسح للسكان على مستوى الأسرة.

النتائج كشفت بياناتنا عن أوجه تشابه بين المرفق وبيانات المسح لبعض المؤشرات (مثل انخفاض الوزن عند الولادة وخدمات الفحص المسبق)، ولكن الاختلافات بالنسبة للآخرين (مثل أمراض الإسهال الحاد لدى الأطفال الذين تقل أعمارهم عن 5 سنوات وفحص ضغط الدم). لقد قدمنا أربع ملاحظات حاسمة (1) تتوفر البيانات على مستوى المرفق ولكن بأشكال مختلفة؛ و(2) قد لا تكون المؤشرات العالمية الراسخة مفيدة دائمًا في الرصد المحلي؛ و(3) يجب تنقيح التعريفات التشغيلية؛ و(4) يعتبر التقسيم الثلاثي والتعليقات من الميدان أمر حيوي.

الاستنتاج نحن نلاحظ أنه بينما يمكن استخدام البيانات لوضع مؤشرات للتقدم، فإن تفسير هذه المؤشرات يتطلب عناية كبيرة. في سبيل السعي لتحقيق التغطية الصحية الشاملة، نحن نعتبر أن ملاحظاتنا حول فائدة بعض المؤشرات الصحية المحددة، ستوفر أفكارًا ذات قيمة للممارسين والمشرفين في وضع آلية لرصد الرعاية الصحية الأولية.

## 摘要

**目的:**

制定初级卫生保健监测框架并生成卫生成效指标列表，以及用于评估 2018-2019 年印度喀拉拉邦卫生改革的实地测试和三角指标。

**方法:**

我们采用改良的德尔菲法 (Delphi) 制定了 23 项指标列表，以监测初级卫生保健。我们使用多阶段群集随机抽样技术，从四个地区群集中分别选取一个地区，然后从这四个地区再分别选取一个家庭和一个初级卫生中心。我们利用机构数据和基于人口的家庭调查对指标进行了实地测试和三角剖分。

**结果:**

我们数据显示，在某些指标（例如低出生体重儿和预检服务）方面，机构和调查数据存在相似性，但在其他指标（如 5 岁以下儿童急性腹泻和血压筛查）却存在差异。我们提出四个关键点 (i) 在机构层面可以获得数据，但格式不同；(ii) 既定的全球指标对本地监测并非一直有用；(iii) 须完善操作定义；(iv) 来自实地的三角剖分和反馈至关重要。

**结论:**

我们发现，虽然可以利用数据来制定进度指标，但对这些指标的解释需要非常谨慎。为实现全民健康覆盖，我们认为，我们针对某些卫生指标效用的观察对从医人员和监督员设立初级卫生保健监测机制非常有价值**。**

## Резюме

**Цель:**

Разработать систему мониторинга первичной медико-санитарной помощи и перечень показателей конечных результатов в отношении здоровья, а также провести тестирование на местах и всесторонне рассмотреть показатели, предназначенные для оценки реформ здравоохранения в штате Керала, Индия, в 2018–2019 гг.

**Методы:**

Авторы использовали модифицированный «дельфийский» метод для разработки перечня показателей, состоящего из 23 пунктов, с помощью которого осуществлялся мониторинг первичной медико-санитарной помощи. Авторы использовали метод многоступенчатой кластерной случайной выборки, чтобы отобрать один район в каждом из четырех районных кластеров, а затем таким же образом выбирали семью и центр первичной медико-санитарной помощи в каждом из четырех районов. Авторы испытали на местах и всесторонне оценили показатели с использованием данных учреждений и анкетирования домохозяйств на уровне популяции.

**Результаты:**

Полученные данные выявили сходство между данными учреждений и данными анкетирования по одним показателям (например, низкая масса тела при рождении и услуги предварительной проверки), но различия по другим показателям (например, острые диарейные болезни у детей младше 5 лет и скрининг артериального давления). Авторы составили четыре важных замечания: (i) данные доступны на уровне учреждения, но в различных форматах; (ii) определенные глобальные показатели не всегда могут использоваться для местного мониторинга; (iii) практические определения требуют уточнения; (iv) всестороннее рассмотрение и обратная связь с мест критически важны.

**Вывод:**

Наблюдения говорят о том, что, хотя данные можно использовать для разработки индексов прогресса, интерпретация этих показателей требует большой осторожности. В достижении всеобщего охвата услугами здравоохранения авторы считают, что их наблюдения о полезности определенных показателей здоровья дадут ценную информацию для практикующих врачей и руководителей при разработке механизма мониторинга первичной медико-санитарной помощи.

## Introduction

Under the thirteenth general programme of work and the triple billion targets,^1^ the World Health Organization (WHO) aims to increase the number of people benefitting from universal health coverage (UHC) by one billion between 2019 and 2023. Central to this effort is the expansion and improvement of primary health-care services.^2^^,^^3^ Progress in achieving UHC can be analysed using the WHO and World Bank’s UHC monitoring framework,^4^^,^^5^ but this requires adaptation to local contexts to ensure health reforms keep pace with targets. Health programmes in India,^6^ as well as the national health policy^7^ and flagship Ayushman Bharat scheme,^8^ are being evaluated in relation to the aims of UHC; various efforts are currently underway at both a national^9^ and state level, notably in Haryana^10^ and Tamil Nadu.^11^

According to National Sample Survey estimates from 2017–2018, morbidity levels in the southern state of Kerala are reportedly four times the national average with disparities by sex and place of residence.^12^ Although the state has made gains in maternal and child health,^13^ it must sustain these gains while addressing the substantial and growing burden of hypertension, diabetes^14^ and cancer;^15^ vaccine-preventable diseases;^16^^,^^17^ and emerging viral infections such as Nipah virus^18^ and severe acute respiratory syndrome coronavirus 2 (SARS-CoV-2).^19^^–^^21^ Kerala has been subject to unregulated privatization and cost escalation,^22^ resulting in persistent inequalities in service access and health attainment between population subgroups.^13^ In 2016, the Government of Kerala announced Aardram, a programme of transformation of existing primary health centres to family health centres;^23^ with increased staffing, these family health centres provide access to a greater number of services over longer opening hours compared with the original primary health centres. 

Apart from the WHO’s monitoring framework,^24^ many countries have done UHC and primary health centre monitoring exercises^25^^,^^26^ alongside independent exercises such as the Primary Health Care Performance Initiative.^27^ However, most of these frameworks are intended for global comparison or decision-making at national levels. The argument for tracking health reforms is clear, but such a monitoring process must be specific to Kerala and local decision-making, while also complying with national and global reporting requirements. Periodic household surveys offer population-level data, but are not frequent enough to inform ongoing implementation decisions. Routinely collected and disaggregated health system data are vital,^28^ but are often marred by quality issues as well as technological and operational constraints.^29^

We began a 5-year implementation research study assessing equity in UHC reforms in January 2018. In our first two phases we aimed to develop a conceptual framework and a health outcome indicator shortlist, followed by validation of these indicators using data from both health facilities and a population-based household survey. We report on the field-testing and triangulation components of this implementation research project, which took place during 2018 and 2019.^30^ We reflect on early lessons from the field-testing and triangulation and, drawing broadly from Ostrom’s institutional analysis and development framework,^31^ we emphasize how monitoring can support learning health systems.^32^^,^^33^ We also discuss how the monitoring of UHC progress requires a flexible approach that is tailored to the local political economy.^34^^–^^36^

## Methods

### Study design

We began with a policy scoping exercise for the state of Kerala in 2018. We then created an 812-indicator longlist from existing primary health-care monitoring inventories,^9^^,^^37^^–^^40^ and undertook an extensive data source and mapping exercise, adapting a process previously conducted in the region.^41^ We applied a modified Delphi process in two rounds, consulting key health system stakeholders of the state (frontline health workers, primary care doctors, public health experts and policymakers), and obtained a shortlist of 23 indicators (available in the data repository).^42^ We then field-tested and triangulated some of the indicators using facility-based data (phases 1 and 2) and a population-based household survey (phase 2). 

### Phase 1: facility data collection 

In phase 1 (December 2018) we selected three family health centres in coastal, hilly and tribal districts (Trivandrum, Idukki and Wayanad, respectively) of the state. We communicated the definitions and logic of the indicators to facility staff, and studied their data-recording methods to synergize our processes with theirs. From these initial steps, we prepared a structured data collection template (available in the data repository)^43^ that we provided to the three family health centres.

### Phase 2: facility data collection

Based on inputs from phase 1 and a second round of consultations with state-level programme officers, we refined the indicator list. In phase 2 (June–October 2019), we used a multistage random cluster sampling technique to generate data related to the indicators at the population and facility level. We applied principal component analysis using Stata version 12 (StataCorp, College Station, United States of America) to data from the latest National Family Health Survey (2015–2016)^44^ to categorize districts into one of four clusters according to health burden and systems performance. Using an open-source list randomizer from random.org, we randomly selected one district from each of the four clusters, and then randomly selected both a primary and a family health centre from each of the four selected districts. The people served by these eight health facilities were the population of interest in our study.

We held on-site meetings with the staff of the eight health facilities and provided them with Excel-based templates (Microsoft Corporation, Redmond, United States of America) to input data for the financial year March 2018–April 2019 (data repository).^43^ Data were sourced from manual registers maintained at facilities. In addition to off-site coordination, we also provided data-entry on-site support to the health staff, visiting each facility at least four times between May and December 2019. We compiled data from the facilities to obtain annual estimates for all health outcome indicators using Excel.

### Phase 2: household survey

Our sample size estimation was based on the proportion of men and women eligible for blood pressure screening under the national primary care noncommunicable disease programme, that is, those aged 30 years or older. We estimated a sample size using routine data reported by the noncommunicable disease division of the Kerala Health and Family Welfare Department (2018–2019), aiming at a precision of 8% at a 95% confidence interval (CI), with a conservative design effect of 2 (i.e. a doubling of the sample). Health facility catchment areas were grouped by wards, also referred to as primary sampling units. Eligible households within a primary sampling unit had at least one member aged 30 years or older. Individual written informed consent was sought from each participant before administration of the survey. We employed and trained staff to collect data using hand-held electronic tablets with a bilingual (English and Malayalam) survey application. The survey, conducted during June–October 2019, included questions on sociodemographic parameters, health outcome indicators (e.g. noncommunicable disease risk behaviours and screening; awareness of components of Aardram and family health centre reform) and financial risk protection (e.g. out-of-pocket expenditure). National Family Health Survey (Round IV) state level weights were applied during analysis.^44^

### Triangulation of phase 2 data

We compared data on selected indicators using Stata and Excel. Since our focus was on how indicators were being understood and reported across facilities, we did not expect indicators to directly correspond between facilities and households, but only to approximate each other.

### Ethics

All components of the study were approved by the Institutional Ethics Committee of the George Institute for Global Health (project numbers 08/2017 and 05/2019).

## Results

We obtained data from 11 health facilities in total (seven family health centres and four primary health centres) during phases 1 and 2. During phase 2, we acquired facility data on indicators from eight health facilities (four family, four primary) jointly serving a population of 273 002 (Table 1). The household survey was undertaken in the catchment areas of these facilities, and we acquired data from a representative sample of 13 064 individuals in 3234 households (Table 1).

**Table 1:** Demographic characteristics of sampled population in field-testing of health-care indicators, Kerala, India, 2018–2019

Characteristics	Family health centres^a^	Primary health centres^a^	Total
Population served by facility	161 317	111 685	273 002
No. male (%)	79 841 (49.5)	54 190 (48.5)	134 031 (49.1)
No. female (%)	81 476 (50.5)	57 495 (51.5)	138 971 (50.9)
Population of households surveyed in catchment area of facilities	5022	8042	13 064
No. households surveyed	1631	1603	3234
No. of people aged ≥ 30 years (%)	3076 (61.2)	4760 (59.2)	7836 (60.0)
No. male (%)	2435 (48.5)	3756 (46.7)	6191 (47.4)
No. female (%)	2582 (51.4)	4279 (53.2)	6861 (52.5)
No. other (%)	5 (0.1)	7 (0.1)	13 (0.1)

Note: Inconsistencies may arise in some values due to rounding.

^a^ Data combined from four different family health centres and four different primary health centres during phase 2 of the implementation study.

We observed both variations between and uniformity in the indicators from health facilities and the household survey (Table 2). In studying these patterns, we made four key observations (Box 1). 

**Table 2:** Triangulation of health-care indicators between populations served by facilities and from household survey, Kerala, India, 2018–2019

Health-care indicator	No. (%) served by facility^a^	No. (%; 95% CI)^b^ from household survey^c^
Pregnant mothers who received all recommended types of antenatal care for most recent live birth in the past year (facility: *n* = 2255; household: *n* = 94)	2479 (109.9)^d^	85 (90.9; 84.7–97.1)
Children < 5 years with diarrhoea in the past year (facility: *n* = 13 552; household: *n* = 900)	912 (6.7)	195 (21.6; 18.1–25.2)
Low birth weight among newborns in the past year (facility: *n* = 2803; household: *n* = 157)	177 (6.3)	17 (11.0; 2.9–19.1)
Pre-check service^e^ by staff nurse, where available (facility: *n* = 146 643; household: *n* = 1152)	98 139 (66.9)	801 (69.5; 59.1–79.9)
Blood pressure screening in the past year (facility: *n* = 273 002; household: *n* = 6367)	39 081 (14.3)^f^	5467 (85.9; 84.5–87.2)^g^
Blood glucose screening in the past year (facility: *n* = 273 002; household: *n* = 6367)	33 965 (12.4)^f^	5254 (82.5; 81.0–84.0)^g^

CI: confidence interval.

Note: Inconsistencies may arise in some values due to rounding.

^a^ The reference period for the facility survey, for which only crude totals reported in registers were available, was April 2018–March 2019.

^b^ Note that numbers used to calculate percentages were provided by Stata to 2 decimal places; we have reported these as whole numbers.

^c^ Weighted using the Fourth National Family Health Survey, India.^44^

^d^ Antenatal care data from one family health centre were considered invalid and excluded from analysis.

^e^ Includes vital sign assessment and case history recording of patients by a staff nurse in a designated area, which is entered into the e-health portal to be available in real time to the consulting physician. Pre-check coverage is now reported as a daily outpatient tally, and is adjusted for returning patients.

^f^ Calculated as number of individuals screened as a proportion of total population served by the facility; age-disaggregated eligible population (≥ 30 years) data were not available.

^g^ Data only obtained from individuals aged ≥ 30 years.

Box 1 **Four observations from field-testing and triangulating health-care indicators, Kerala, India, 2018–2019**

Observation 1: Data are available at the facility level, but in varying formats and platforms meant for different purposes; digitization may improve this situation.

Observation 2: Established global indicators may not be useful or interpreted as intended in a local context, and may need to be adapted.

Observation 3: Operational definitions, thresholds for interpretation and processes of routine data collection must be refined for older indicators and developed for newly introduced indicators.

Observation 4: Triangulation and feedback from the field level, with qualitative input from local actors, remains vital, particularly for chronic diseases.

Note: Tested indicators are given in Table 2.

First, the method of reporting our indicators varied between facilities, even although all raw data required to calculate selected indicators were present in manual registers. In the case of indicators related to national programmes (e.g. reproductive, child health and tuberculosis-related indicators), data were uploaded directly to national digital portals without any analysis at the facility level; officers responsible for data compilation and analysis exist only at the district level. Feedback from facility staff included requests for adequate training on new or revised reporting systems, and clarification of their role. This situation may improve with the complete digitization of health records under Kerala’s e-health programme.

Our second observation is that there exist two problems with the globally recommended indicators: (i) manual routine data reporting at the facility level may be inadequate to construct the global indicator precisely; and (ii) globally relevant data may not be considered relevant to the periodicity (monthly) or level (facility) of review. From the facility-level data, the coverage of antenatal care reported by family health centres was 109.9% (2479/2255); in household surveys, full coverage of antenatal care was observed for 90.9% (85/94) of eligible women (Table 2). Here, antenatal care refers to women aged 15–49 years having a live birth in the past year and receiving four or more antenatal check-ups, at least one tetanus toxoid injection, and iron and folic acid tablets or syrup for at least 100 days as numerator. The coverage rate is calculated from a denominator of the total number of women aged 15–49 years who had a live birth in the past year, which requires retrospective verification of antenatal coverage. However, in some facilities, the antenatal care coverage indicator was calculated using the previous year’s number of deliveries plus 10% as the denominator, and the number of pregnant women who had received antenatal care as the numerator. It was therefore not always clear that the data from any particular individual were included in both the numerator and denominator and, with a target as the denominator, coverage could surpass 100%. Practitioners noted the disconnect between monthly target-based reporting and annual retrospective measurement. 

Our third observation is that definitions and reporting that reflect actual health-provision patterns require to be standardized; otherwise, discrepancies will be observed between data sets. For example, the indicator for acute diarrhoeal diseases among children younger than 5 years was 6.7% (912/13 552) according to facility records; however, a prevalence of more than 3 times this percentage (21.6%; 195/900; 95% CI: 18.1–25.2) was reported in the household survey (Table 2). Several chronic care indicators, newly introduced as part of the introduction of family health centres, also showed discrepancies. For instance, the percentage of people screened for blood pressure and blood glucose was 85.9% (5467/6367; 95% CI: 84.5–87.2) and 82.5% (5254/6367; 95% CI: 81.0–84.0), respectively, according to the household survey; however, facility data yielded a screening coverage of 14.3% (39 081/273 002) and 12.4% (33 965/273 002), respectively. These observed discrepancies could be the result of: (i) a large proportion of the population being screened for certain conditions and risk factors in the private health sector; (ii) the fact that age-disaggregated data are not available at the facility level; and/or (iii) while the national guidelines recommend population-based screening of all adults aged 30 years and older, Kerala has introduced opportunistic screening for blood pressure among all aged 18 years and older. Discrepancies were not observed for all indicators, however; results for low birth weight and pre-check services were similar between the two data sets.

Our fourth observation is that such triangulation exercises, as well as obtaining feedback from health workers, programme managers and administrators, are vital for accurate assessment of UHC coverage.^45^ A major problem reported by staff and officials is that health facility data are usually just a tally of patient visits, which is simple to produce, as opposed to the actual number of (potentially repeat) patients receiving care or services. State officials have been encouraging a move towards electronic health records to generate more precise indicators, but adoption and integration of these will only be possible when the technology itself is better aligned to facility-level process flows, requiring user inputs, investment and time. Other issues raised include: the need for appropriate staff (including temporary contractual staff) training in programme guidelines and reporting requirements; the need for clarity in definitions of treatment (e.g. chronic disease patients may be advised to modify lifestyle factors, which would be missed if treatment monitoring included only those prescribed medication); and the availability of free or subsidised tests relevant to disease control that are reflected in monitoring indicators, particularly for chronic care (e.g. glycated haemoglobin tests for diabetes care^46^) at the primary health centre level. 

## Discussion

As already observed in India and other low- and middle-income countries,^29^ our results indicate that any approach to improving or monitoring the quality of health-care must be adaptable to local methods of data production and reporting, while ensuring that emerging concerns of local staff are considered. Although validity checks are a staple of epidemiological and public health research, such triangulation processes in health systems are infrequent. The Every Newborn-BIRTH study was a triangulation of maternal and newborn health-care data in low- and middle-income countries,^47^ and some smaller-scale primary-care indicator triangulation exercises have been undertaken by India’s National Health Systems Resource Centre.^48^^,^^49^ While there exists a variety of approaches to monitoring primary health-care reforms,^30^ we consider the most appropriate to be the generation (and modification, if necessary) of indicators from routine data, and their triangulation with household survey data.^49^

Increasingly, routine data are being digitized to improve accessibility and interpretation, as is the case in Kerala. Useful considerations when introducing digital health interventions in low- and middle-income countries are intrinsic programme characteristics, human factors, technical factors, the health-care ecosystem and the broader extrinsic ecosystem.^50^ Our observations demonstrate the continuous and complex interplay between these characteristics; the real value of selected indicators may also be determined by how staff understand and interpret them.

Our study had several limitations. Our indicator selection using the Delphi method could have undergone additional rounds, but we considered it more important to get the monitoring process underway and reduce the burden on health workers. Some facility-based information could not be acquired due to the additional health department burden of flood relief and Nipah outbreak management in the state. Our household survey sample was the population aged 30 years and older, resulting in undersampling for other indicators being field-tested (e.g. newborn low birth weight). An increase in sample size could allow a more precise estimation of all indicators. Finally, the reference periods for the facility data and the household survey did not directly overlap; a timed sampling should be undertaken in the future to improve the precision of triangulation.

Observing the utility of indicators in practice is a key first step in the move towards UHC, requiring investment and commitment. Using indicators, standards and other forms of technology, which are easy to adopt, can be problematic because we amplify certain aspects of the world while reducing others.^51^ Our examination of family health centre reforms cautions that, while data can be used to develop indices of progress, interpretation of these indicators requires great care precisely because of the way they are related to powerful decisions around what constitutes success or failure, who will receive recognition or admonition and, ultimately, the legacy of Aardram reforms. We anticipate that our observations will contribute to health-care reforms in low- and middle-income countries, such as the use of field triangulation to enhance the accountability and relevance of global health metrics.^36^ If such activities are carried out in constructive partnerships with state stakeholders and do not introduce unfeasible costs to the system, they may contribute to a sustained and reflexive monitoring process along the path to UHC.
